# Spatial and Temporal Distribution of West Nile Virus in Horses in Israel (1997–2013) - from Endemic to Epidemics

**DOI:** 10.1371/journal.pone.0113149

**Published:** 2014-11-17

**Authors:** Karin Aharonson-Raz, Anat Lichter-Peled, Shlomit Tal, Boris Gelman, Daniel Cohen, Eyal Klement, Amir Steinman

**Affiliations:** 1 Koret School of Veterinary Medicine, The Robert H. Smith Faculty of Agriculture, Food and Environment, The Hebrew University of Jerusalem, Rehovot, 76100, Israel; 2 School of Public Health, Sackler Faculty of Medicine, Tel Aviv University, Tel Aviv, 39040, Israel; 3 Kimron Veterinary Institute, Bet-Dagan, 50250, Israel; CEA, France

## Abstract

With the rapid global spread of West Nile virus (WNV) and the endemic state it has acquired in new geographical areas, we hereby bring a thorough serological investigation of WNV in horses in a longstanding endemic region, such as Israel. This study evaluates the environmental and demographic risk factors for WNV infection in horses and suggests possible factors associated with the transition from endemic to epidemic state. West Nile virus seroprevalence in horses in Israel was determined throughout a period of more than a decade, before (1997) and after (2002 and 2013) the massive West Nile fever outbreak in humans and horses in 2000. An increase in seroprevalence was observed, from 39% (113/290) in 1997 to 66.1% (547/827) in 2002 and 85.5% (153/179) in 2013, with persistent significantly higher seroprevalence in horses situated along the Great Rift Valley (GRV) area, the major birds' migration route in Israel. Demographic risk factors included age and breed of the horse. Significantly lower spring precipitation was observed during years with increased human incidence rate that occurred between 1997–2007. Hence, we suggest referring to Israel as two WNV distinct epidemiological regions; an endemic region along the birds' migration route (GRV) and the rest of the country which perhaps suffers from cyclic epidemics. In addition, weather conditions, such as periods of spring drought, might be associated with the transition from endemic state to epidemic state of WNV.

## Introduction

West Nile virus (WNV) is a member of the *Flavivirus* genus in the Flaviviridae family [1]. Since the virus was first isolated from the blood of a febrile woman in Uganda in 1937 [2] it has become recognized as one of the most widely distributed flaviviruses in humans as well as in horses. West Nile virus infection was first reported in the Mediterranean basin, in both Egypt and Israel, in the early 1950s and cases with severe neurological manifestations in humans were reported for the first time in 1957 in Israel [3]. In horses, WNV encephalomyelitis was first recorded in the Middle East in 1959 in Egypt [4]. Antibodies to WNV were detected in horses in Israel already in the 1960s [5], but outbreaks were not reported during the next 3 decades. Until the mid-1990's, WNV was not considered an important differential diagnosis for equine encephalomyelitis disease. However, several large scale, well documented outbreaks among equine populations raised the awareness to this disease. West Nile virus encephalomyelitis outbreaks were reported in Morocco in 1996 [3], in Italy in 1998 [6], in France in 2000 [7] and in Israel in 2000 [8]. The dramatic appearance of WNV in New York City (NYC) area in 1999 and its subsequent spread in North America largely contributed to the overall focus the disease acquired. During the decade since its first discovery in NYC (between 1999 and 2012), a total of 37,008 cases of West Nile fever and 16,196 cases of neuroinvasive West Nile disease were reported in humans in the US, with incidence of neuroinvasive disease between 0.1 and 1.0 per 100,000 [9]. Not only is the incidence of West Nile in horses (∼700 per 100,000) substantially higher than in humans [10], the disease in horses seems to be more severe; over 25,000 horses in USA have been affected since 1999, with 33% case-fatality and 40% of survivors with neurological sequelae. In humans, 4–9% case fatality was recorded and 30% of encephalitis survivors with sequelae [11]. Another interesting difference observed was that when data was compared between 904 confirmed human cases gathered from the European Centre of Disease Prevention and Control and 200 confirmed equine cases gathered from the World Organisation for Animal Health during 2010, it was noticed that equine morbidity started three weeks later than humans' [12].

West Nile virus imposes great threat on human and animal health not only by emerging in new geographical areas but also as it causes epidemics in endemic areas. Despite the presence of WNV in most of the United States already a decade ago and its spread to all regions of the continental US throughout this decade [13], WNV epidemic activity was demonstrated in 2012, in which more human disease cases were reported nationally than any year since 2003. This outbreak resulted in 2,873 cases of neuroinvasive disease and 286 human deaths [14], [15]. Available data suggest that the increased incidence of WNV disease in 2012 was not likely caused by genotypic changes in the circulating virus strains and it was suggested that a number of inter-related factors, including weather, abundance of birds that maintain the virus, abundance of mosquitoes that spread the virus, and human behaviour are all contributors to the disease epidemics [16]. WNV has caused sporadic outbreaks in humans, horses and birds throughout many of the warmer regions of Europe for at least 20 years. However, currently, WNV appears to be expanding its geographical range in Europe and causing increasing numbers of outbreaks associated with human morbidity and mortality. The increase in the number of WNV cases in the last few years in central and southern Europe is likely due to the introduction of a lineage 2 WNV to central Europe (Hungary) in 2004 [17]. After a few years of adaptation, this virus strain has been heavily spreading first within Hungary and to the eastern part of Austria [18], and thereafter through the Balkan states to Greece [19], causing a significant outbreak of human West Nile neuroinvasive disease. In the last 3 years, in addition to the Balkan area, large human epidemics and WNV human involvement was also detected in Italy, Hungry, the Russian Federation and Ukraine [20], [21]. Israel serves as a crossroad for bird migration between Africa and Euroasia, and therefore a major focus of attention during the global spread of WN fever (WNF).

Israel has been considered endemic to WNV since the first recognized human WNF outbreak in 1951. Thereafter, decades of small, intermittent outbreaks were reported in 1952, 1953, 1957 and 1980. However, in 2000 Israel experienced its largest recorded outbreak affecting hundreds of people [22], dozens of horses [8] and several flocks of geese [23]. During the following decade, between 2000 and 2012, nearly 1,400 cases of human WNF were reported. Identified cases were fewer between 2001 and 2004 but greatly increased in 2005 and stayed consistently high since then [24]. Previous studies that raised assumptions for the causes of re-emergence of WNV in endemic areas focused mainly on human data. Biotic factors raised include past immunity and increased in awareness among physicians and more frequent request for laboratory testing, while abiotic factors discussed include climatic parameters (i.e. temperature, relative humidity and precipitation) and environmental drives (i.e. bird migration) [20], [25]. Since horses serve as incidental hosts to WNV, similar to humans, characterizing the spatial and temporal distribution of disease occurrence in horses may shed light on the overall prevalence of WNV in an endemic region such as Israel. Therefore, the objectives of this study were to identify the seroprevalence and force of infection of WNV in horses in Israel for a period of more than a decade, between 1997 until 2013, and to characterize demographic and environmental risk factors associated with WNV occurrence. In addition, factors associated with transition from endemic to epidemic state were also examined.

## Materials and Methods

### Blood samples

Blood samples were collected from clinically healthy horses distributed throughout Israel, in order to determine seroprevalence of WNV in the years 1997, 2002 and 2013. In 1997, 290 horses, located in 12 farms were sampled. In 2002, 827 horses located in 52 farms were sampled and in 2013, 179 horses, located in 16 farms were sampled. All of these horses have not been vaccinated to WNV (vaccination of horses began only after 2002 and in 2013 vaccination status was gathered from the owners). Blood collections were performed under owners' consent and all surveys involving blood collection were approved by the Internal Ethics Review Committee of the Koret School of Veterinary Medicine, The Hebrew University. Farms sampled were located throughout Israel according to the estimated geographical distribution of horse farms in the county, with higher farms density in the center, followed by the north and finally in the south of Israel. The horse population in Israel is estimated to be between 20,000–30,000 horses. Most horses live for many years in the same farm, but traveling between farms occurs occasionally, mainly for competition purposes or for trail rides.

### Serum preparation, serum neutralization test (SN) and competitive enzyme-linked immunosorbent assay (cELISA)

Blood samples were collected from the jugular vein of each horse into sterile serum-separating tubes and centrifuged at 3000 rpm for 8 minutes. Sera were collected and stored at −80°C until further analysis. Seroprevalence of WNV was detected using the SN method for the 1997, 2002, and 2013 serum samples and the ELISA method for the 2013 serum samples.

For the SN test, Vero cells were grown on Eagle's medium with 10% fetal calf serum (Bio-logical Industries Ltd. Beit Haemek, Israel) and 1% antibiotic solution, including penicillin, streptomycin, and amphotericin (Bio-logical Industries Ltd. Beit Haemek, Israel). The cells were maintained in a humidified atmosphere at 37°C in 5% CO_2_ and were used for growing virus stocks and virus neutralization assay. Double dilutions from 1/10 to 1/1280 of 10 µl of each serum sample were made in 96 well plates (F 96 cell culture plates, Nunc A/S, Roskilde, Denmark) in Eagle's medium containing 50 µl of the challenge virus WN98 (genbank AY033388) [26] at a concentration of 10^3.3^ TCID50 [27]. The dilutions were allowed to stand for 1 h at room temperature. Vero cells were then added to each well, and the plates were placed in a 37°C incubator (5% CO_2_) for 4 days, when they were inspected microscopically for cytopathic effect (CPE). The neutralizing antibody titer of the serum was expressed as the reciprocal of the highest dilution at which complete neutralization of the CPE was observed. Each titer was then calculated as the percentage of the titer of a known positive control that was checked on each assay.

Immunoglobulin G (IgG) levels in the 2013 serum samples were also determined using a commercial ELISA kit (ID Screen West Nile Competition Multi-species, IDvet Innovative Diagnostics, France), according to the manufacturer's instructions. Samples presenting an S/N percentage (OD sample divided by OD negative control) ≤40 were considered positive. Optical density values were obtained using an automatic plate reader. Seroprevalence analyses relied on the SN results for the 1997 and 2002 samples and on the ELISA results for the 2013 samples.

### Collection of demographic and environmental data

**Figure 1:**
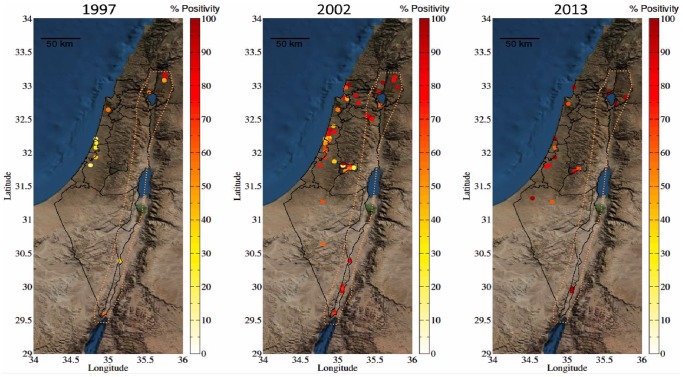
Spatial distribution of WNV seroprevalence in horses in Israel during 1997, 2002 and 2013. Circles inside the dashed orange marking represent farms located in the Great Rift Valley (GRV) area.

**Figure 2:**
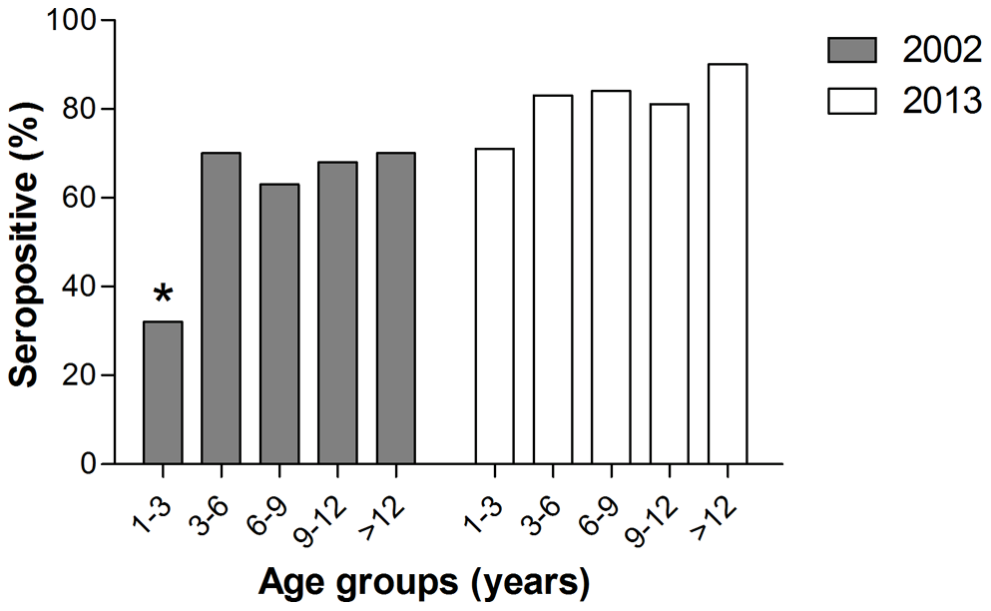
The association of WNV seroprevalence with age in 2002 and 2013. No significant differences were observed in the seroprevalence between the different age groups except for the youngest age group (1–3 yr) in 2002, in which a significant lower seroprevalence was observed. *P<0.05.

During blood collections information on age, gender, colour, breed and geographical location was recorded for every subject. Age was analysed both as a continuous variable and as a categorical one (age groups: 0–3 yr, 3–6 yr, 6–9 yr, 9–12 yr 12–15 yr and >15 yr). The most complete data was gathered for 2002, soon after the large WNV outbreak, and therefore this year was chosen to represent the demographic risk factors analysis. The demographic variable of the breed of the horse was recorded for 808 out of the 827 horses that participated in 2002; with 134 Quarter horses (16.6%), 15 Appaloosa (1.9%), 104 (12.9%) Cross breeds, 27 (33.5%) Local breeds, 40 (5%) Ponies, 18 (2.2%) Thoroughbreds, 139 (17.2%) Warmbloods and 87 (10.8%) Arab horses. For the purpose of geographical/environmental analyses Israel was divided to two zones: (1) Great Rift Valley (GRV) zone (including the Golan Heights, the Steppe/Arava zone, parts of the Galilee; north/valley/sea of Galilee and the Jordan Valley) representing the dominant route of birds migration in Israel (Figure 1) and (2) the rest of the country.

### Calculation of force of infection using age specific data

Force of infection (FOI), a measure of the incidence of infection in a susceptible population, specifying the rate at which susceptible individuals acquire an infection, was estimated using a likelihood method, by maximizing the following likelihood function [28]:
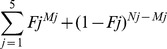


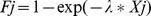
Where j is the index for the 5 age groups (0–3 yr, 3–6 yr, 6–9 yr, 9–12 yr, >12 yr), Nj is the total number of horses sampled in age group j, Mj is the number of horses that are WNV seropositive in age group j, Xj is the average age in age group j, and λ is the assumed FOI. Maximizing the likelihood function was performed using the solver add-in of Microsoft EXCEL 2010.

### Precipitation levels in epidemic vs. non-epidemic years

Information on monthly precipitation (mm) was gathered from the Israel Meteorological Service. Data were collected from all listed weather stations distributed throughout Israel in the years 1997–2009. Spring and autumn/winter precipitation levels were determined by averaging the amount of precipitation for the months of March–May and October–December at all stations, respectively.

In a period of a decade, between 1997–2007, two years demonstrated a prominent increase in incidence rate of WNV in humans: 2000 and 2005 [24]. Precipitation data from these years (2000 and 2005) as well as one year prior to each of these years (1999 and 2004) (“increased WNV incidence”) was compared with the precipitation data from the rest of the years; 1997, 1998, 2001, 2002, 2003, 2006, 2007 (“steady WNV incidence”).

### Statistical analyses

An interrater reliability analysis using the Kappa statistic was performed to determine consistency among the ELISA and the Serum Neutralization results of the 2013 samples.

Seroprevalence and its 95% confidence interval were calculated for each category of each demographic variable (breed, gender, age, and colour) for 2002, as well as for the different geographic zones for 1997, 2002, 2013. Statistical significance of the differences in prevalence between these various groups was assessed by the two-sided Chi-square test. A logistic regression model was constructed with variable inclusion in the model performed by the forward likelihood ratio method. Demographic and geographical variables were included in the model according to statistical significance, and p value for change in the model likelihood ratio after insertion of each additional variable was tested. In each such iteration, a p value of <0.05 was considered as a criterion for inclusion in the model. After each inclusion, variables that were already in the model and showed a p value >0.1 were excluded.

Independent-samples t-test analyses were used to compare average precipitation in years with increased incidence of human WNV in Israel (1999, 2000, 2004 and 2005) to the rest of the years throughout the decade of 1997–2007. These analyses were performed for the spring and autumn/winter precipitation volumes. Differences were considered significant at p value <0.05. All statistical analyses were performed using SPSS 17.0 software.

## Results

### Seroprevalence and age-specific force of infection of WNV during 1997, 2002 and 2013

Out of the 290 horses tested in 1997, 113 (39%) were seropositive for WNV, thereafter in 2002, 547/827 (66.1%) horses were seropositive and finally, in 2013 out of 179 horses, 153 (85.5%) were found seropositive for WNV. The above seroprevalence was determined using the SN method for the 1997 and 2002 samples. Kappa coefficient showed an almost perfect agreement between the ELISA results and the SN results of the 2013 samples, with 86% agreement (p value <0.001), and hence further analyses for the 2013 samples were based on the ELISA results.

Seventy one horses were tested both in 1997 and in 2002 and all of them remained in the same geographical location during this time period. Among these 71 horses, 31 (44%) had neutralizing antibodies to WNV already in 1997 and all 31 remained positive when sampled again during 2002. Of the 40 horses that were negative in 1997, 16 (40%) seroconverted during these 5 years and 24 (60%) remained negative during this period. The force of infection using age specific data was 12.3% and 22.3% in 2002 and 2013, respectively. In 2013, a high seroprevalence of 71%–90% was observed in all age groups; 1–3 yr, 3–6 yr, 6–9 yr, 9–12 yr and >12 yr, with no significant differences in seroprevalence between the different age groups. In 2002, no significant differences were observed in the seroprevalence between the different age groups except for the youngest age group (1–3 yr), in which a significant lower seroprevalence was observed (Chi square: odd ratio = 3.62–4.96, p value <0.01) (Figure 2). Not enough data on the age of the horses was collected in 1997 in order to perform adequate statistical analyses.

### Geographical/Environmental risk factors for spatial distribution of WNV during 1997, 2002 and 2013

Univariate analysis demonstrated significantly higher seroprevalence in horses located in the GRV area than in those located out of the GRV area in all three years (66.1% vs. 31.6% in 1997, 84.8%vs. 59.7% in 2002 and 100% vs. 78.9% in 2013) (Table 1). This was also the case when logistic regression analysis was performed on the 2002 results (Table 2) as well as on the 1997 and 2013 results (data not shown).

**Table 1:** Univariate analyses for the association of WNV seroprevalence in horses in Israel and the geographical location of the horses along the Great Rift Valley - GRV (1997, 2002, 2013).

		Positive/total (%)	OR	95% CI	P
**1997 GRV**	**Rift valley area**	41/62 (66.1)	4.23	2.25–8.08	<0.01
	**No Rift valley area**	72/228 (31.6)	REF		
**2002 GRV**	**Rift valley area**	179/211 (84.8)	3.77	2.48–5.86	<0.01
	**No Rift valley area**	368/616 (59.7)	REF		
**2013 GRV**	**Rift valley area**	56/56 (100)	1.268*	1.16–1.39	<0.01
	**No Rift valley area**	97/123 (78.9)	REF		

OR: odds ratio; CI: confidence interval; P: p value; REF: reference variable; GRV: Great Rift Valley.

*Prevalence ratio.

**Table 2:** Logistic regression model for the association of WNV seroprevalence in horses in Israel with demographic (age, gender, breed, colour) and geographical/environmental (Great Rift Valley - GRV) risk factors in 2002 (n = 802).

		Exp(B)	S.E.	P value	95% CI
**AGE**		1.044	0.016	<0.007	1.044–1.012
**BREED**	Quarterhorse	REF			
	Arabian	0.692	0.307	0.231	0.379–1.264
	Warmblood	0.304	0.279	<0.001	0.176–0.525
	Thoroughbred	1.123	0.575	0.84	0.364–3.466
	Pony	0.359	0.385	0.008	0.169–0.764
	Local	1.502	0.251	0.105	0.919–2.454
	Cross	1.481	0.318	0.217	0.794–2.764
	Appaloosa	0.470	0.562	0.179	0.156–1.413
**GRV**		2.773	0.224	<0.001	1.789–4.298

Exp(B): odds ratio (the exponentiation of the B coefficient); S.E.: standard error; CI: confidence interval; GRV: Great Rift Valley; REF: reference variable.

Calculation of FOI using age-specific data for the years 2002 and 2013 revealed higher rate of infection in horses located in the GRV area as compared to those located outside of the GRV area (22.8% in GRV vs. 9.8% outside the GRV and 50% in the GRV and 16.6% outside the GRV in 2002 and 2013, respectively).

### Demographic risk factors for WNV in 2002

In the univariate analysis, significant association was detected between WNV seroprevalence and age, colour, gender and breed of the horses. A significantly lower seroprevalence was observed in the youngest age group (0–3 yr), in the dark colour group (black/dark brown/dark grey) as compared to the light colour group (grey/light appaloosa), in males, and in the Arabian, Warmblood, Pony and Appaloosa breeds as compared to the cross breeds (Table 3). Gender showed close association with GRV area as significantly more female horses were sampled in the GRV area (Pearson Chi-Square: 15.891, p value <0.01). Entering all above demographic and geographical location factors into a logistic regression model, age, breed and GRV were found to significantly associate with seroprevalence to WNV; age showed positive association, Warmblood breeds and Ponies were significantly lower in seroprevalence than the Quarter horse breed and horses located in the GRV had significantly higher seroprevalence to WNV than horses located outside this area (Table 2).

**Table 3:** Univariate analysis for demographic risk factors associated with WNV seroprevalence in horses in Israel in 2002 (n = 827).

		Positive/total (%)	OR	95% CI	P
**AGE (YEARS)**	**0–3**	12/38 (31.6)	0.20	0.09–0.43	<0.01
	**3–6**	128/184 (69.6)	1.02	0.66–1.57	1.00
	**6–9**	120/191 (62.8)	0.73	0.48–1.10	0.11
	**9–12**	89/131 (67.9)	0.91	0.57–1.47	0.73
	**12+**	193/276 (69.9)	REF		
**COLOUR**	**Paint**	16/25 (64)	0.75	0.29–2.04	0.498
	**Black/Dark brown/Dark grey**	54/102 (52.9)	0.47	0.28–0.80	<0.01
	**Bay/Chesnut**	313/469 (66.7)	0.85	0.58–1.23	0.415
	**Palamino/Buckskin**	24/32 (75)	1.26	0.51–3.45	0.678
	**Grey/Light appalloosa**	140/199 (70.4)	REF		
**GENDER**	**Female**	280/396 (70.7)	1.48	1.10–2.01	<0.01
	**Male**	267/431 (61.9)	REF		
**BREED**	**Arabian**	51/87 (58.6)	0.36	0.18–0.71	<0.01
	**Warmblood**	55/139 (39.6)	0.17	0.09–0.31	<0.01
	**Thoroughbred**	13/18 (72.2)	0.66	0.19–2.63	0.534
	**Pony**	19/40 (47.5)	0.23	0.10–0.54	<0.01
	**Appaloosa**	7/15 (46.7)	0.22	0.06–0.80	<0.01
	**Quarterhorse**	94/134 (70.1)	0.59	0.31–1.13	0.101
	**Local**	212/271 (78.2)	0.91	0.49–1.63	0.780
	**Cross**	83/104 (79.8)	REF		

OR: odds ratio; CI: confidence interval; P: p value; REF: reference variable.

Age was not recorded for 7/827 horses. Breed was not recorded for 19/827 horses.

### Average precipitation in spring and autumn/winter of 1997–2007

**Figure 3:**
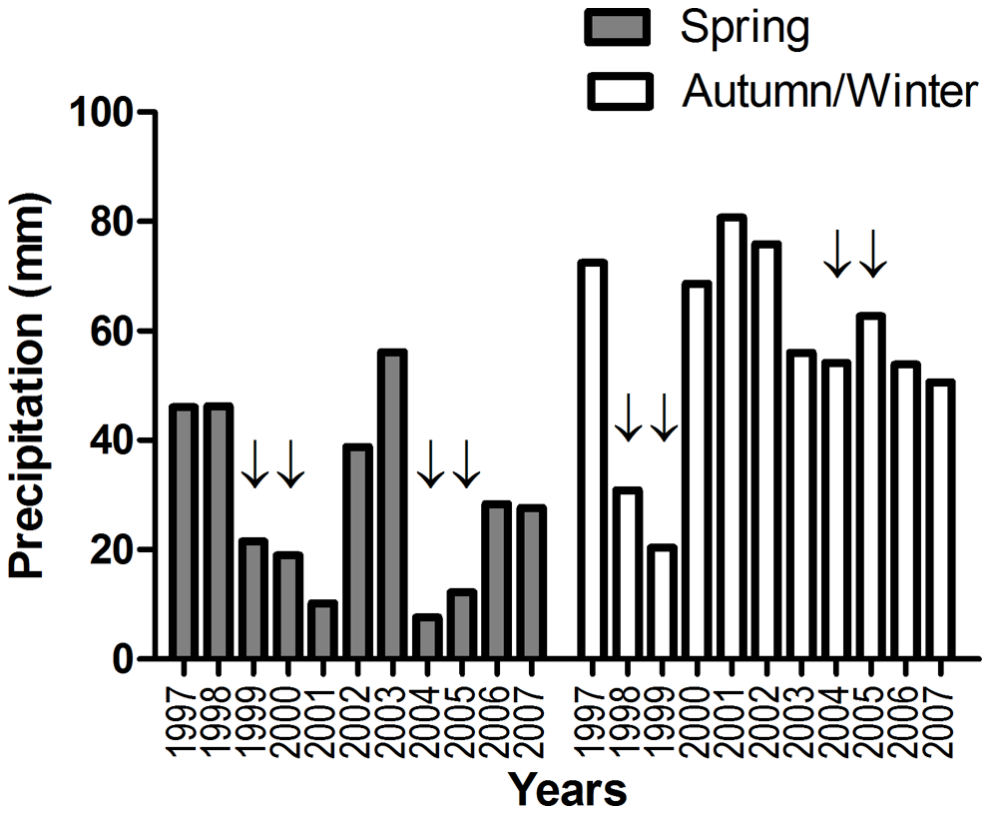
Average spring and autumn/winter precipitation (mm) in the years 1997–2007. Arrows represent years with documented increased human WNV incidence. Between 1997–2007, the average spring precipitation (mm) was significantly lower (P<0.05) in the group of years belonging to the “increased WNV incidence” (arrows), as compared to the rest of the years. No differences in average autumn/winter precipitations were noted.

During the decade from 1997–2007, the average spring precipitation (mm) was significantly lower in the group of years belonging to the “increased WNV incidence” which include the years 1999/2000 and 2004/2005, as compared to the rest of the years (belonging to the “steady WNV incidence”) (15.08 mm versus 36.18 mm, respectively, p value = 0.03). However, no differences in average autumn/winter precipitations were noted between these two groups (51.45 versus 60.06, respectively) (Figure 3).

## Discussion

### Seroprevalence, force of infection and factors associated with transition from endemic to epidemic state

Although WNV was shown to circulate in Israel since the 1960's, it has been receiving growing attention only in the last decade since the 2000 WNF outbreak among humans and horses. In a serological survey performed during 1959–1960, a 35% seroprevalence to WNV was detected in horses in Israel [29]. Following three decades, as well as a documented WNV outbreak in humans in 1980 [30], a 39% seroprevalence in horses was detected in our study for the year 1997. The findings in the current study indicate that the growing attention to the disease in the equine population in Israel in the last decade is not only due to the increase in the number of horses in Israel and improved veterinary care and services which enabled the identification of the virus, but also to a true surge in virus occurrence among horses in this region. An increase in seroprevalence was detected from 39% seropositivity in 1997, just prior to the WNF outbreak, to 66.1% in 2002, following the large outbreak, and remained high in 2013 with 85.5% seroprevalence. In addition, of the horses that were negative in 1997, 40% seroconverted during a 5-years period. If the infection rate throughout the period between 1997 and 2002 was constant, this demonstrates a rate of infection of 8% per year. Nevertheless, the true infection rate cannot be estimated due to the large scale outbreak in 2000. The high occurrence of WNV in horses in Israel in the last decade is also demonstrated by the high FOI detected in this study, with 12.3% and 22.3% FOI in 2002 and 2013, respectively. It could be argued that the increase in seroprevalence demonstrated in this study between 2002 and 2013 could be a result of different antibody detection methods, as the ELISA method used for the 2013 samples is considered more sensitive than the SN method used for the 2002 samples [31]; however, it has previously been shown that WNV-neutralizing test was able to confirm 42 out of 49 ELISA-positive horse sera (86%) [32]. Furthermore, in addition to the ELISA method, serum neutralization (SN) test was also employed on the 2013 samples, as was previously performed to evaluate WNV seroprevalence in horses in Germany [33], and excellent agreement was demonstrated between the two detection methods with a kappa of 86.4% (p value <0.001), further supporting the validity of the ELISA results and the conclusions drawn from them.

The increase in seroprevalence of WNV in horses in the last decade is in accordance with human reported cases of WNV in Israel [24]. Although following the 2000 outbreak a decrease in incidence rate was documented, this lasted only until 2005 in which a substantial increase in incidence occurred during that year and remained constant until 2012 [24]. Cyclic epidemics of endemic arboviral diseases are recognized phenomena and have previously been addressed. For example, Eastern equine encephalitis (EEE) virus epidemics occur in the northern regions of the United Stated and tend to cluster over 2 or more consecutive years, separated by periods of dormancy. Several factors were suggested to be responsible for re-emergence of EEE and it has been suggested that although historical risk factors have included above-average rainfall, and mild winters, records show a trend toward milder winters and hotter summers, marked by extremes in both precipitation and drought in the northeastern United States [25], [34].

Ambient temperature was constantly shown to play an important role in WNV transmission by affecting the growth rates of vector populations, the interval between blood meals, and viral replication rates [20]. Furthermore, during the WNV outbreak in Israel in 2000, the minimum temperature was found to be the most important climatic factor that encouraged earlier appearance of disease [35]. Nonetheless, the contribution of precipitation to the transmission and spread of WNV is more complex and controversial. Heavy rainfall during spring may increase standing water resources at the beginning of the hot season and therefore increase the vectors natural habitats. In contrast, during drought conditions, the reduced water flow creates stagnant water pools which become richer in the organic material ideal for breeding mosquitoes. This may encourage birds to circulate around small water holes and thus increase the interactions with mosquitoes. These discrepancies regarding the role of precipitation in WNV transmission raised the assumption that the patterns of disease incidence may change over large geographic regions (i.e. tropical vs. Mediterranean regions), depending on differences in the ecology of mosquito vectors [12]. Numerous studies describe the association of drought conditions with WNV human infection in the United States [36]. Some identified inverse relationship between total annual rainfall levels of the previous year and the relative risk of human WNV [37], as well as negative correlation between WNV infection in mosquitoes and the previous year's precipitation [38]. Others report dry spring and summer conditions during the same year an increased risk of human WNV infection was observed [25], [39]. Alternatively, widespread drought conditions in the spring, followed by a wetter summer was also linked with increased probability of human WNV cases [40]. Similar correlation between drought periods and outbreaks of WNV has been documented in Europe. In 1996, a significant WNV outbreak occurred in Romania coinciding with prolonged drought conditions (May through October) and excessive heat (May through July) [41]. In the summer of 1999, a large WNV outbreak occurred in Russia following a drought period [42]. In the current study, precipitation data gathered from the Israel Meteorological Services revealed significant lower spring precipitation level during the years with increased WNV human cases (2000 and 2005) and one year prior to them (1999 and 2004) as compared to the rest of the years from 1997 to 2007, supporting the notion of association between WNV epidemics and a prior dry spring season. Similar to the periodic increased incidence of WNV cases in Israel, the United States experienced a resurgence of WN neurologic disease in 2012 with the highest level of disease in the last decade [43]. Interestingly, the resurgence of disease followed a period of nearly 4 years of extremely low incidence rate similar to the 4 years that preceded the increased human incidence in 2005 in Israel. The low incidence rate prior to a resurgence of the disease, observed both in Israel and the US, may suggest the involvement of decreased herd immunity in the incidental host (humans, horses) as well as in the main amplifying host (birds) [44]. Perhaps both the dry springs as well as the decreased immunity in the years prior to the outbreaks contributed, together with many other interrelated biotic and abiotic drivers (i.e. temperature, relative humidity and bird migration), to the WNV epidemics that occurred in humans in Israel during 2000 and 2005.

### Spatial and temporal characterization of geographical/environmental and demographic risk factors for WNV seroprevalence

Locations where large outbreaks as well as sporadic cases of WNV occurred in the United States have varied from year to year, demonstrating their focal nature, and is probably in part a result of variation in the immune state of the susceptible population. In contrast, via spatial and temporal investigation of WNV prevalence, the current study demonstrates a consistent geographical disparity of seroprevalence to WNV among horses in Israel, with a persistent higher seroprevalence in horses located throughout the Great Rift Valley (Syrian-African Rift Valley) area than in horses located outside of this area (in all three years; 1997, 2002 and 2013), as well as higher FOI in horses located in the GRV area in 2002 and 2013 as compared to those located outside of this area. The association between bird migration routes and WNV dispersion worldwide and specifically in Israel has been well documented [20], [23], [45]–[47]. Although the whole country of Israel is considered the crossroad for bird migration between Africa and Euroasia, and therefore a major focus of attention during the global spread of WNF, three principal routes exist inside the country, the dominant one along the Great Rift Valley [48]. Based on our findings, we suggest being more cautious with the WNV endemic terminology for Israel, and perhaps a more precise description of the region ought to consider only the Great Rift Valley regions as WNV endemic regions (probably due to the migrating birds passing along, introducing the virus to the region), while all other regions of Israel perhaps suffer from cyclic epidemics of WNV activity every few years. Work et al. (1955) pioneered in dividing the distribution of WNV prevalence around the Nile delta region into “endemic”, “transition” and “non-endemic” areas [49]. This division was shown both for wild birds as well as human seroprevalence. Only few children and adults were shown to have antibodies for WNV in the northern portion of the Nile delta along the Mediterranean coast (“non-endemic area”, 2% and 30%, respectively) as compared to higher percentage of seropositive children and adults in villages located in the southern part of the delta (“endemic area”, 70% and 95%, respectively), and intermediate level in the transitional area, located further inland [50]. Great variation in seroprevalence was also observed in horses in different areas of sub-Saharan Africa, with low seroprevalence in the east of the Sahelian area (9%) and in sub-Sahelian area (3–30%) and highest seroprevalence in the western and central parts of the Sahelian area (92%–97%) [51].

Demographic variables that were shown to associate with seroprevalence to WNV were age and breed of the horses. Previous studies demonstrated the association of age with seroprevalence to WNV in endemic areas. Prior to the 2000 WNF human epidemic in Israel, seroprevalence was shown to increase with age, as in healthy soldiers aged 18–20 years a lower seroprevalence was noted (7.0%) compared to the older age group (40–55 years) with 41.9% seropositivity to the virus [52]. This increase in seroprevalence with age was also observed in Iran, another WNV endemic region, in which a difference of 5 years in horses' age corresponded to a change of seropositivity odds by a factor of 1.3 [53]. Surprisingly, in the current study, when we divided the horses into age groups in the two sampling periods in this study (i.e. 2002 and 2013), seroprevalence to WNV was not shown to differ much between the different age groups. In 2013, the very high overall seroprevlance and FOI could have masked differences between groups as from a very young age horses were largely exposed to the virus. In addition, the great outbreak that occurred in 2000 hit all age groups equally and therefore in 2002 no differences were found between the different age groups, except for the youngest age group (1–3 yr), which include foals that were not yet born during the outbreak and therefore the seroprevalence of that group was significantly lower. The reason for the lower WNV seroprevalence found in our study among Ponies and Warmblood horses, as compared to other breeds, is unknown and might indicate different preference of mosquitoes to different breeds of horses, due to variables such as density of hair and differences in sweat composition. In addition, grooming and anti-pesticides regimes are most likely stricter in the Warmblood breeds since they are mostly used for competitions in Israel while the Quarter horses and cross breeds are used mostly for pleasure riding. The higher seroprevalence detected in the Quarter horses in our study is in accordance with a study performed in Florida, USA, in which Quarter horses were the most commonly affected breed [13], and with a study performed in Saskatchewan, Canada, which indicated that most of the WNV clinical cases were light horse breeds and most were used for pleasure riding [54]. However, as opposed to the above study, in which dark coloured horses were most commonly affected with WNV [54], univariate analysis in the current study demonstrated higher seroprevalence in the lighter colours (grey/light appaloosa; 70.4%) as compared to the darker colours (black/dark brown/dark grey; 52.9%). Though, since these results were not repeated in the logistic regression model, the above findings might be due to association between colour and breed.

## Conclusions

The extensive serological survey in horses, throughout duration of more than a decade, covering periods before (1997) and after (2002, 2013) the largest documented WNF outbreak in Israel (2000), permitted the investigation of the spatio-temporal dynamics of WNV in a longstanding endemic region such as Israel. We hereby demonstrate that the 3 serene decades since the 1960's, in which constant seroprevalence to the virus in horses was observed, were interrupted in the last decade when an increase in exposure to the virus was detected from 39% seroprevalence just prior to the 2000 WNF outbreak to 66.1% and 85.5% seroprevalence in 2002 and 2013, respectively. Demographic variables such as age and breed were found to significantly associate with seroprevalence to WNV. But more prominently, environmental factors (geographical location and precipitation amounts), and more specifically the geographical location of the horse along the Great Rift Valley, the main route of birds migration in Israel, was found to be of great impact on the likelihood of WNV exposure throughout the years investigated. Humans, similar to horses, serve as incidental hosts to WNV and therefore may share common epidemiological aspects regarding disease occurrence and spread. Interestingly, weather conditions, and more specifically rainfall conditions, such as periods of spring drought, together with lower incidence rate in the population, were shown to precede transition from endemic state to epidemic state of WNV observed in humans in Israel during the years 2000 and 2005.
